# Treatment of Neuralgia

**Published:** 1871-10

**Authors:** G. J. Kollock

**Affiliations:** Savannah, Ga.


### AN OBSTINATE CASE OF NEURALGIA TREATED.

*Report of the Treatment of an Obstinate Case of Neuralgia of the Uterus in the Female Ward of the Sav. P. H. and Hospital.*

BY G. J. KOLLOCK, M. D., Savannah, Ga.

Sophia Johnson, age 23, a native of Germany, married, and the mother of one child, was admitted into the Female Ward of the Savannah Hospital, on the 15th July, for the treatment of a uterine and vaginal mucus discharge. Upon examination, the case was pronouned one of chronic cervical endometritis.

Her general health being not materially impaired, I determined upon the employment of constitutional measures without any resort to local remedies; at intervals of every few days, she complained of acute uterine pains, which were relieved by anodynes. On the 12th of August she was seized with violent spasmodic pains in the hypogastric region, recurring at intervals of every five minutes; this was accompanied with an irritable condition of the stomach and vomiting. After a thorough inquiry and examination into the case I was satisfied that the trouble was referable to the uterus, and I at once endeavored to alleviate her suffering. At 8 p. m., I administered 1-12 gr. Atropine, hypodermically, over the region of the uterus, and at the same time I ordered an assifœtida enema, and ½ gr. sulph. morphia interally; these measures gave her no relief, and at 12, m., I administered ⅓ gr. sulph. morphia, hypodermically.

On the morning of the 13th August, I found her still suffering and without having obtained any relief, whatever. At 9 a. m., I again administered ⅓ gr. sulph. morphia, hypodermically, and an enema of castor oil and assafœtida. I also, applied a hot mustard poultice over the hypogastric region. The enema produced a free evacuation of the bowels, but the uterine pain continued with unabated severity; I ordered an anodyne injection of ½ gr. sulph. morphia, which produced no effect; the continuance of the pain and the severity of its nature, brought on an extremely nervous condition, and fearing that this might tend to some permanent affection of the brain, I administered chloroform by inhalation. By this means I hoped, at least, to bring about a temporary cessation of the agony under which the patient was suffering, and to allow the nervous system some relaxation and tone to recuperate. She slept about two hours, and on awakening the pains were ushered in with as much severity as before.

I then ordered a vaginal suppository of belladonna and opium, and again administered 1-12 gr., hypodermically, no appreciable effect followed, and at 10 p. m., I again resorted to the use of chloroform. She slept until 4½ a. m. the next morning, and when she awoke the neuralgic pain in the uterus again returned, with as much severity as previously, August 14th. The irritability of the stomach having, in a measure, subsided, I gave a small quantity of beef tea, which was retained, some time after this, I gave 30 grs. hydrate chloral, which was repeated after an interval of three or four hours ; this also, was retained upon the stomach, but produced no sleep, and failed entirely to bring about that relief which I had hoped it would. Finding that all the measures which I had employed produced no effect, I again resorted to the inhalation of cloroform, which was repeated at intervals of six or eight hours, and which, at last, brought about a temporary relief.

Upon the suggestion of Dr. Harriss, who was consulted in the case, I determined to apply leeches to the cervix, and on the 15th of August, I made a vaginal examination with the speculum. The os uteri was patulous and the cervix presented a puffed and swollen appearance, with engorgement of the vessels. After the preliminary step of closing the os with a plug, I applied six leeches, five of which took very readily, while the sixth manifested no disposition to bite. Soon after the application of the leeches, the blood flowed freely, which it continued to do for five or six hours, and which I encouraged. On the afternoon of the 15th, the pains had almost ceased and my patient felt much relieved. On the morning of the 16th, they had ceased entirely, and since that time she has continued to improve. During the whole continuance of this case the pulse remained perfectly quiet and regular, ranging only from 72 to 76 or 78.

I have thus been minute in the details of the application of the remedies used, to show that, so far as I could observe, none of them produced any appreciable effect, except the one resorted to, at last—viz : the application of the leeches.

It may be thought that a severe neuralgic affection of this character would, after a time, wear itself out, and that this might have been the case in this instance, but after a close and careful attendance upon the patient from the commencement of her suffering up to the close, I have no hesitancy in expressing the opinion that relief was due entirely to the application of the leeches, and that all the other remedies proved utterly useless. Had the application of the leeches failed, I would have tried electricity directly to the womb, which I have seen produce a soothing effect.

